# The HMG-CoA reductase inhibitor, simvastatin, exhibits anti-metastatic and anti-tumorigenic effects in ovarian cancer

**DOI:** 10.18632/oncotarget.5834

**Published:** 2015-10-16

**Authors:** Jessica E. Stine, Hui Guo, Xiugui Sheng, Xiaoyun Han, Monica N. Schointuch, Timothy P. Gilliam, Paola A. Gehrig, Chunxiao Zhou, Victoria L. Bae-Jump

**Affiliations:** ^1^ Division of Gynecologic Oncology, University of North Carolina, Chapel Hill, NC, USA; ^2^ Department of Gynecologic Oncology, ShanDong Cancer Hospital & Institute, Jinan University, Jinan, P.R. China; ^3^ Lineberger Comprehensive Cancer Center, University of North Carolina, Chapel Hill, NC, USA

**Keywords:** simvastatin, ovarian cancer, HMGCR, invasion, apoptosis

## Abstract

Ovarian cancer is the 5th leading cause of cancer death among women in the United States. The mevalonate pathway is thought to be a potential oncogenic pathway in the pathogenesis of ovarian cancer. Simvastatin, a 3-hydroxy-3-methyl-glutaryl-CoA reductase (HMGCR) inhibitor, is a widely used drug for inhibiting the synthesis of cholesterol and may also have anti-tumorigenic activity. Our goal was to evaluate the effects of simvastatin on ovarian cancer cell lines, primary cultures of ovarian cancer cells and in an orthotopic ovarian cancer mouse model. Simvastatin significantly inhibited cellular proliferation, induced cell cycle G1 arrest and apoptosis, and caused cellular stress *via* reduction in the enzymatic activity of HMGCR and inhibition of the MAPK and mTOR pathways in ovarian cancer cells. Furthermore, simvastatin induced DNA damage and reduced cell adhesion and invasion. Simvastatin also exerted anti-proliferative effects on primary cell cultures of ovarian cancer. Treatment with simvastatin in an orthotopic mouse model reduced ovarian tumor growth, coincident with decreased Ki-67, HMGCR, phosphorylated-Akt and phosphorylated-p42/44 protein expression. Our findings demonstrate that simvastatin may have therapeutic benefit for ovarian cancer treatment and be worthy of further exploration in clinical trials.

## INTRODUCTION

Ovarian cancer is the 5^th^ leading cause of cancer death among women with an estimated 21,290 new cases diagnosed in the United States in 2015 and 14,180 deaths [1]. The majority of these cancers are found in later stages. Initial treatment typically includes a surgical staging or a debulking procedure followed by adjuvant chemotherapy [2]. Overall survival for advanced ovarian cancer is poor, with less than 40% of patients surviving at 5 years [2, 3]. Thus, there is a great need to develop more effective treatments for this deadly disease.

A growing body of literature supports obesity as a significant risk factor for the development of ovarian cancer [4–7]. In addition, obesity has been linked to worse outcomes and higher mortality from this disease [4, 7, 8]. The metabolic mechanisms for these phenomena are not fully understood. It is thought that obesity leads to elevated insulin signaling, inflammation, an increased availability of lipids, and changes in adipokine signaling that results in the conversion of normal epithelial cells to invasive tumor cells. Our recent results showed that the metabolic effects of obesity have been shown to promote ovarian cancer pathogenesis and aggressiveness in a genetically engineered mouse model of serous ovarian cancer [5].

Simvastatin belongs to the statin class of drugs that are widely used in the treatment of hypercholesterolemia. It is an effective and well-tolerated drug with few side effects. Statins inhibit 3-hydroxy-3-methyl-glutaryl coenzyme A reductase (HMGCR), which is an enzyme that is required for the synthesis of mevalonate [3]. HMGCR is essential for the cellular synthesis of cholesterol and a variety of non-steroid isoprenoid derivatives involved in cell proliferation, differentiation and survival. *In vitro* and *in vivo* studies suggest that simvastatin inhibits cancer cell growth by inducing apoptosis and inhibiting cell cycle progression through a variety of cell signaling pathways [9–13]. Pre-clinical studies highlight the ability of statins to decrease cancer cell proliferation, invasion and metastasis by inhibiting the synthesis of cholesterol, required for cancer growth [14–16]. Phase II clinical trials have demonstrated that some patients may benefit from simvastatin combined with other chemotherapeutic agents [17].

Observational studies have shown a meaningful reduction in ovarian cancer risk with long-term use of statins [18, 19]. In a recent meta-analysis of the effect of statins in gynecologic cancers, the use of statins was associated with a significant 21% risk reduction (RR = 0.79; 95% CI, 0.64–0.98) in the incidence of ovarian cancer [20]. This risk reduction persisted with long term statin use >5 years (RR = 0.48; 95% CI, 0.28–0.80) [20]. Despite these findings, there have been limited *in vitro* studies and no *in vivo* studies on the effects of simvastatin on ovarian cancer tumor growth. Thus, we sought to investigate the effect of simvastatin on cell proliferation, apoptosis, cellular stress, adhesion and invasion in ovarian cancer cells and in an orthotropic mouse model of ovarian cancer. Our results indicate that simvastatin demonstrates promise as a targeted agent for ovarian cancer.

## RESULTS

### Simvastatin inhibited cell growth and decreases HMGCR activity

**Figure 1:**
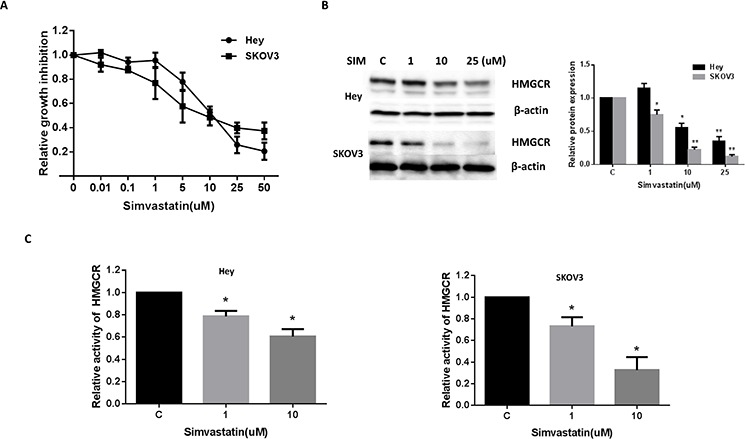
Simvastatin inhibited the growth of ovarian cancer cells and HMGCR activity Hey and SKOV3 cells were cultured for 24 h and then treated with varying concentrations of simvastatin in 96 well plates for 72 h. Cell proliferation was assessed by MTT assay **A.** The effect of simvastatin on its target, HMGCR, was examined by Western blot analysis. Simvastatin treatment resulted in a dose-dependent decrease in expression of HMGCR protein in both cell lines **B.** HMGCR activity in ovarian cancer cells was measured *via* HMGCR Assay. Treatment with simvastatin for 24 h resulted in a dose-dependent decrease in HMGCR activity in both the Hey and SKOV3 cell lines **C.** Each experiment was performed three times. C in graphs refers to control. (*p < 0.05, **p < 0.01).

The effect of simvastatin on cell proliferation was examined in the ovarian cancer cell lines, Hey and SKOV3. The cells were exposed to varying doses of simvastatin for 72 h. As shown in Fig. 1A, simvastatin effectively inhibited cell proliferation in a dose-dependent manner in both ovarian cancer cells. The mean IC_50_ value for each of these cell lines was approximately 10 uM and 8 uM for Hey and SKOV3 cells, respectively. In order to ensure that simvastatin had an inhibitory effect on its molecular target, we examined HMGCR protein expression and activity in both cell lines after exposure to varying doses of simvastatin (1, 10 and 25 uM) for 24 h. A significant decrease in protein expression and activity of HMGCR was seen in the Hey and SKOV3 cells (Fig. 1B–1C), co-incident with inhibition of proliferation.

### Simvastatin induced cell cycle arrest in G0/G1 and apoptosis

**Figure 2:**
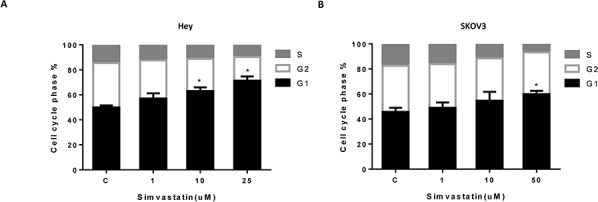
Simvastatin induced cell cycle G1 arrest in ovarian cancer cells The Hey **A.** and SKOV3 **B.** cell lines were treated with the indicated doses of simvastatin (1–25 uM) for 24 h. Cell cycle analysis was performed by Cellometer. Simvastatin markedly induced cell cycle G1 arrest in both cell lines in a dose dependent manner. Each experiment was performed three times. C in graphs refers to control. (*p < 0.05, **p < 0.01).

**Figure 3:**
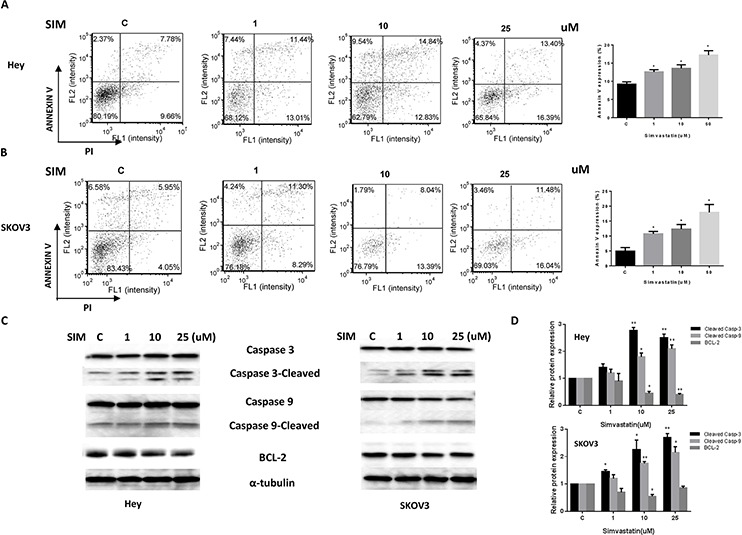
Simvastatin increased apoptosis in ovarian cancer cells The Hey **A.** and SKOV3 **B.** cell lines were cultured for 24 h and then treated with simvastatin at different doses for 24 h. Apoptosis was examined by Annexin V assay in Cellometer. Caspase-3, Caspase-9 and BCL-2 were determined by Western immunoblotting after exposure to simvastatin for 10 h or 24 h **C** and **D.** Each experiment was performed three times. C in graphs refers to control. (*p < 0.05, **p < 0.01).

To evaluate the underlying mechanism of growth inhibition by simvastatin, the cell cycle profile was analyzed after treating the Hey and SKOV3 cells with varying doses (1–25 uM) of simvastatin for 24 h. Simvastatin treatment resulted in G0/G1 cell cycle arrest and reduced S phase in a dose-dependent manner in the cells (Fig. 2A and 2B). To further confirm whether the growth inhibition of ovarian cancer cells was related to apoptosis, we evaluated the apoptotic effect of simvastatin on Hey and SKOV3 cells by Annexin-V FITC stain analysis, which detects the phospholipid phosphatidylserine (PS) translocation from the inner (cytoplasmic) leaflet of the cell membrane to the external surface in very early apoptotic cells. As shown in Fig. 3A and 3B, after treatment of the cells with simvastatin at the indicated concentrations for 24 h, the percentage of early apoptotic cells increased in a dose-dependent manner in both cell lines. We next determined whether the mitochondrial apoptosis pathway, which leads to caspase activation and induces cell death, was involved in simvastatin-induced apoptosis in ovarian cancer cells. We treated both cells with the indicated concentration of simvastatin for 10 hours, and cleaved caspase 3 and cleaved caspase 9 proteins were determined by Western blotting using antibodies that specifically detect the cleave forms of the caspases. We observed a dose-dependent increase in expression of cleaved caspase proteins in both cell lines in response to simvastatin (Fig. 3C and 3D). In addition, we found simvastatin reduced BCL-2 protein expression in a dose dependent manner after treatment with simvastatin for 24 hours in Hey and SKVO3 cells (Fig. 3C and 3D). These result suggest that inducing mitochondrial apoptosis and cell cycle G1 arrest may be major mechanisms to inhibit cell proliferation in simvastatin treated ovarian cancer cells.

### Simvastatin increased levels of intracellular ROS and resulted in DNA damage

**Figure 4:**
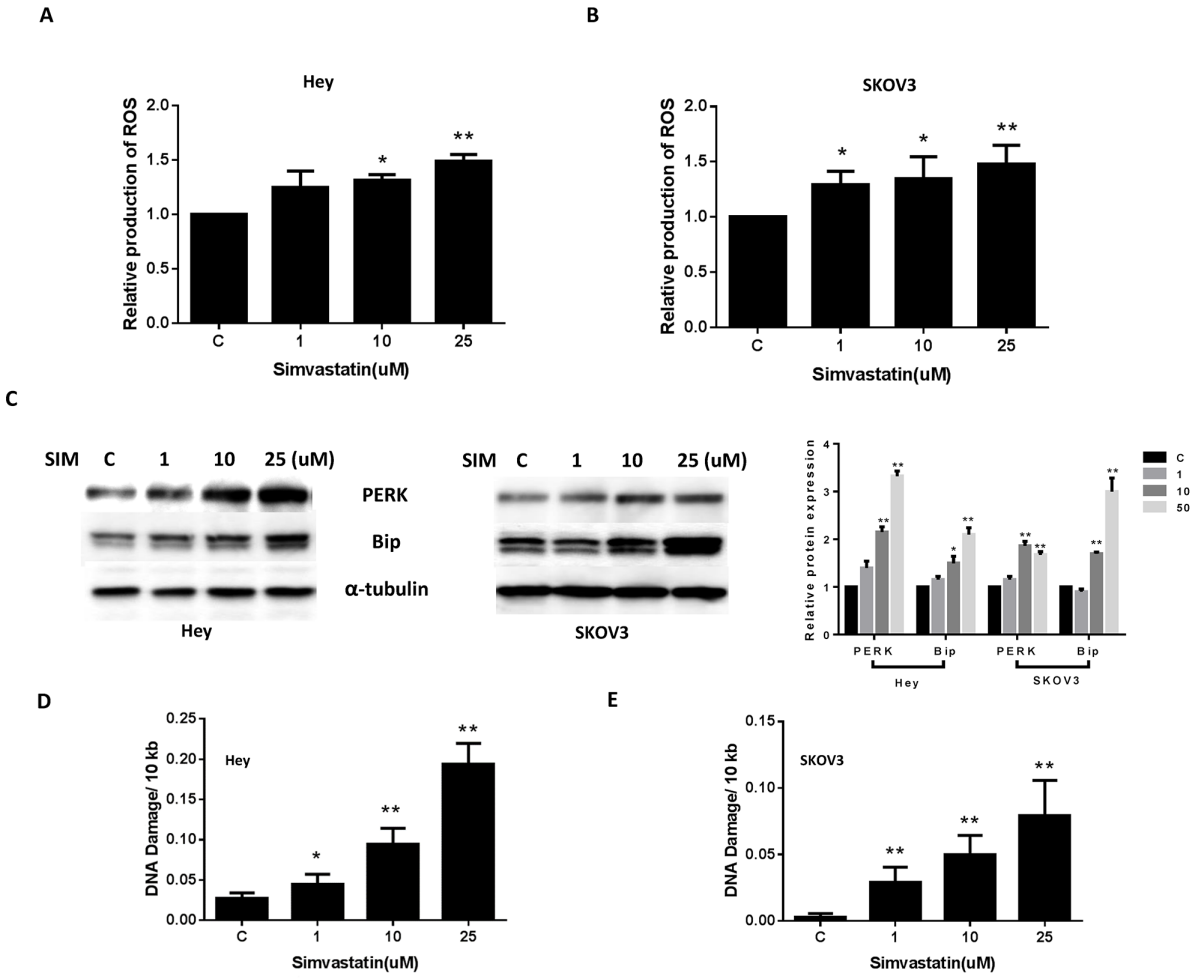
Simvastatin caused cellular stress and DNA damage in ovarian cancer cells The Hey **A.** and SKOV3 **B.** cell lines were treated with simvastatin at different concentrations for 18 h and reactive oxygen species (ROS) level was determined using DCFH-DA dye on a plate reader. PERK and Bip were determined by Western immunoblotting after exposure to simvastatin for 24 h **C.** DNA damage in the Hey **D.** and SKOV3 **E.** cell lines was analyzed by QPCR assay after treatment with simvastatin for 24 h. Each experiment was performed three times. (*p < 0.05, **p < 0.01). C in graphs refers to control.

Reactive oxygen species (ROS) have been implicated in the cellular response to stress and are involved in mediation of apoptosis *via* mitochondrial DNA damage [20]. To investigate the involvement of oxidative stress in the anti-proliferative effect of simvastatin, intracellular ROS levels were examined by using the ROS fluorescence indicator DCFH-DA [17]. As seen in Fig. 4A and 4B, treatment with simvastatin (1–25 uM) for 18 h significantly increased cellular ROS production in a dose-dependent manner in the Hey and SKOV3 cells. We next examined the changes of markers for endoplasmic reticulum (ER) stress after 24 h treatment of simvastatin in both cell lines. Western blotting showed simvastatin induced PERK and Bip protein expression in a dose dependent manner, which is further evidence of ER stress induction by simvastatin (Fig. 4C). Given that the production of peroxides and free radicals induced in oxidative stress can damage several cell components including nuclear and mitochondrial DNA, we evaluated DNA damage by qPCR and found that simvastatin induced DNA damage in a dose-dependent manner in both the Hey and SKOV3 (Fig. 4D and 4E) cells after 24 h of treatment. These results indicate that an increase in ROS and DNA damage might also be involved in the anti-tumorigenic effects of simvastatin in ovarian cancer cells.

### Simvastatin inhibited cell adhesion and invasion

**Figure 5:**
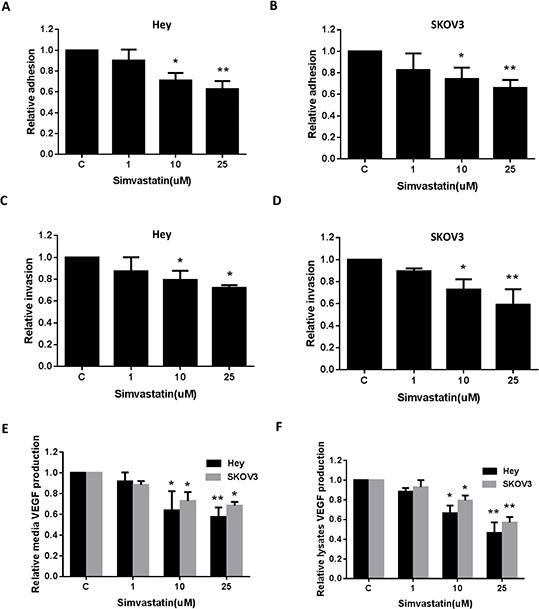
Simvastatin reduced on adhesion and invasion in ovarian cancer cells The Hey **A, C.** and SKOV3 **B, D.** cell lines were cultured for 24 h and then treated as indicated with simvastatin in a laminin coated 96 well plate for 2 h to assess adhesion or a BME coated 96 transwell plate for 24 h to assess invasion, respectively. The data represents relative inhibition in each cell line. VEGF was measured by ELISA assay in culture media **E.** and cell lysates **F.** after a 36 h exposure to simvastatin. Each experiment was performed three times. (*p < 0.05, **p < 0.01). C in graphs refers to control.

Adhesion and invasion are crucial to the evolution of metastatic disease. In order to determine the effect of simvastatin on invasive ability of ovarian cancer cells, *in vitro* laminin adhesion assay and transwell invasion system were employed. Incubation of the Hey and SKOV3 cells with simvastatin (1, 10 and 25 uM) for 2 h showed significant inhibition of cell adhesion (Fig. 5A and 5B). Simvastatin significantly blocked ovarian cancer cell invasion after 24 h of treatment as determined by transwell invasion assay (Fig. 5C and 5D). Inhibition of cell adhesion and invasion was dose-dependent in the both cells. Since vascular endothelial growth factor (VEGF) is a key mediator of angiogenesis and invasion in cancer, we next sought to determine the direct effects of simvastatin on production of VEGF in both cell lines. Simvastatin significantly reduced the levels of VEGF in culture media and cell lysates after 36 hours of treatment (Fig. 5E and 5F). These results suggest that simvastatin may function to inhibit adhesion and invasion in ovarian cancer cells as well as inducing apoptosis, cell cycle arrest, cellular stress and reducing angiogenesis.

### Effect of simvastatin on the AKT/mTOR and MAPK pathways

**Figure 6:**
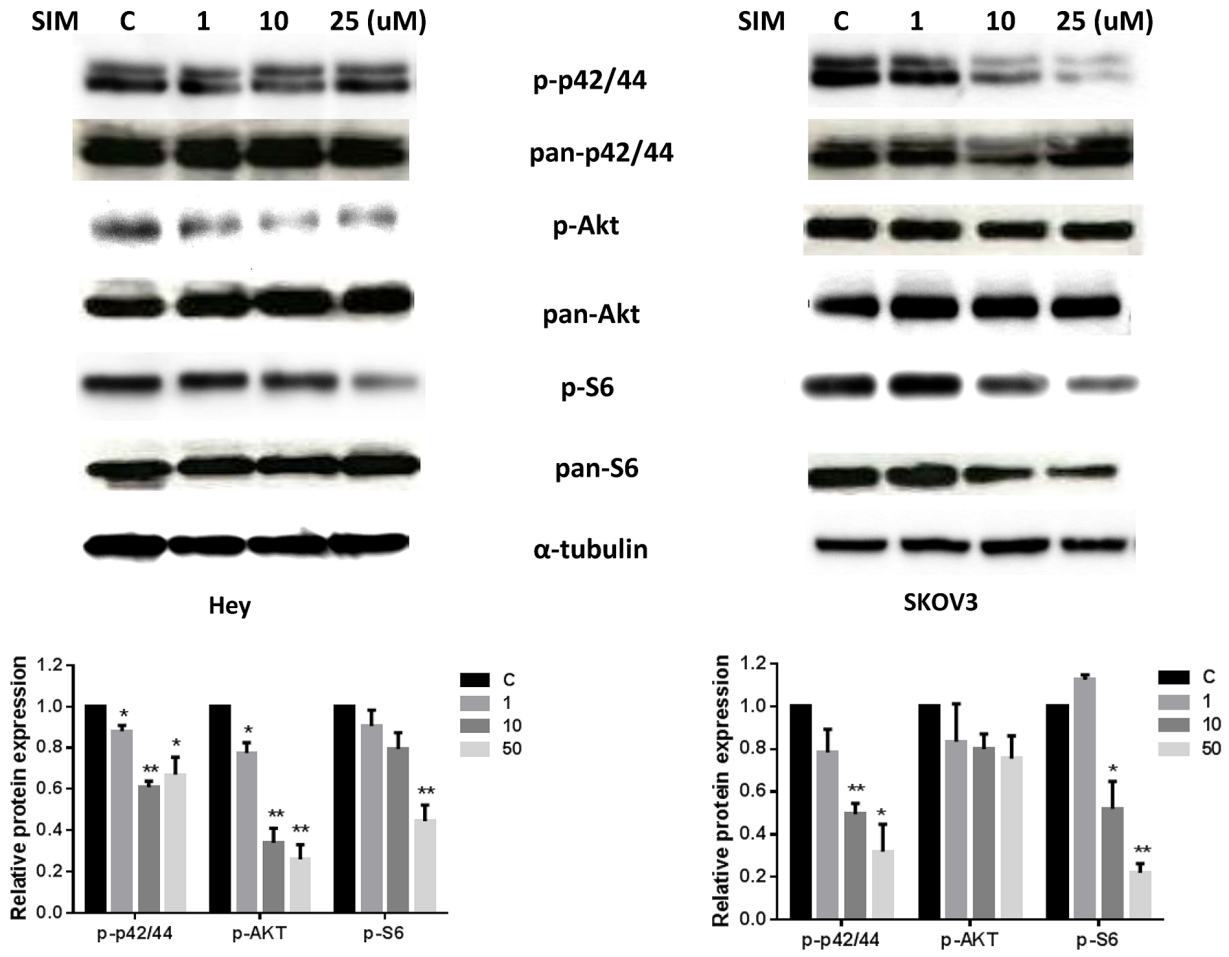
Simvastatin inhibited MAPK and AKT/mTOR pathways in ovarian cancer cells Hey and SKOV3 cells were treated with simvastatin at different doses for 24 h. Phosphorylated-p42/44, phosphorylated-AKT and phosphorylated-S6 were assessed by Western blotting. Simvastatin inhibited the activity of MAPK and AKT/mTOR pathways in Hey and SKOV3 cells. Each experiment was performed two times. (*p < 0.05, **p < 0.01).

It is well known that activation of the extracellular signal regulated kinase (ERK)/mitogen-activated protein kinase (MAPK) and AKT/mammalian target of rapamycin (AKT/mTOR) pathways play a crucial role in the control of cell growth and survival in ovarian cancer and inhibition of these pathways leads to the inhibition of ovarian cancer growth [21, 22]. To investigate the mechanisms underlying the inhibition of cell growth by simvastatin, we characterized the effect of simvastatin on these signaling pathways. Simvastatin reduced phosphorylation of p42/44 (ERK1/2) in a dose-dependent manner in both the ovarian cancer cell lines, within 24 h of exposure (Fig. 6). We then evaluated the effect of simvastatin on the AKT/mTOR/S6 pathway. Western blotting showed that simvastatin decreased phosphorylation of AKT and S6 in both cell lines in a dose dependent manner after 24 h treatment (Fig. 6). These data suggest that simvastatin may exert its anti-tumor activity *via* inhibition of MAPK and AKT/mTOR/S6 pathways.

### Simvastatin decreased tumor growth in an orthotopic mouse model of ovarian cancer

**Figure 7:**
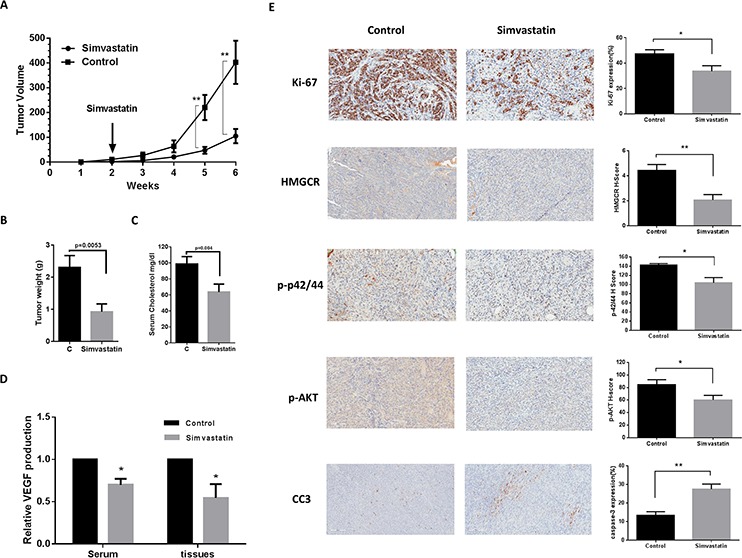
Simvastatin reduced tumor growth of orthotropic xenografts of serous ovarian cancer M909 cells were injected into left side of the ovarian bursa of 6–8 week old mice. When the tumors reached ~50 mm^3^ (approximately 12 days after injection), the mice were treated with placebo or 3 mg/kg simvastatin once a day for 4 weeks. Tumor volume **A.** and weight **B.** were recorded during or after 4 weeks treatment. Simvastatin reduced serum cholesterol in the mice **C.** VEGF was measured by ELISA assay in mouse serum and tumor tissues **D.** The changes of Ki-67, cleaved caspase 3, HMGCR, phosphorylated-AKT and phosphorylated-p42/44 were assessed by immunohistochemistry in ovarian tumor tissues **E.** (*p < 0.05. **p < 0.01).

To validate the anti-tumorigenic potential of simvastatin *in vivo*, we utilized an orthotopic serous ovarian cancer mouse model by injecting M909 cells into the ovary bursa of female mice [23]. The mice were treated with simvastatin (intraperitoneal injection, 3 mg/kg/day) or placebo (saline) for 4 weeks after twelve days of injection with M909 cells. Tumor growth during the treatment was monitored by palpation twice a week. When tumors reached a certain size (more than 0.4–0.5 cm), we measured the tumor size by caliper. During the treatment, the mice showed tolerance to simvastatin injections and maintained normal activities. Regular twice-weekly measurements yielded no changes in blood glucose or weight (data not shown). After 4 weeks of treatment, the mice were euthanized, and the ovarian tumors were removed, photographed, and weighed. A substantial reduction in tumor growth, tumor weight was found in the simvastatin group in comparison with the placebo group (Fig. 7A and 7B). We also found that simvastatin significantly reduced cholesterol concentration in mouse serum (Fig. 7C), indicating that mevalonate pathway is able to impact the inhibition of tumor growth induced by simvastatin *in vivo*. Furthermore, simvastatin was shown to reduce VEGF production in mouse serum and tumor tissues by ELISA assays (Fig. 7D).

To further confirm the anti-tumorigenic activity and mechanism of simvastatin *in vivo*, the expression of Ki-67 (marker of cell proliferation), cleaved caspase 3 (marker of apoptosis), phosphorylated-AKT, phosphorylated-p42/44 and HMGCR was evaluated by immunohistochemistry. Consistent with our results *in vitro*, the expression of phosphorylated-AKT, phosphorylated-p42/44 and HMGCR was reduced in the mice treated with simvastatin but not in the placebo-treated mice. Ki-67 was significantly reduced following simvastatin treatment compared to the controls, whereas simvastatin increased the levels of cleaved caspase 3 in the treated mice (Fig. 7E). These results confirm that simvastatin inhibits ovarian tumor growth *via* activation or inhibition of critical signaling pathways involved in proliferation and metabolism.

### Simvastatin inhibited proliferation of ovarian cancer cells derived from patients

**Figure 8:**
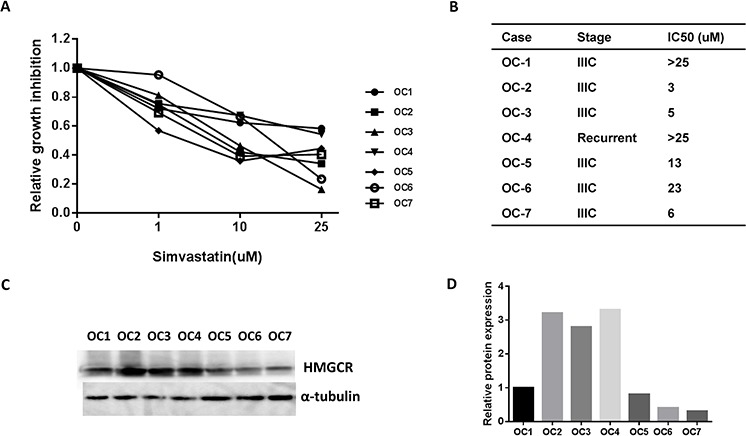
Simvastatin decreased cell proliferation in primary cultures of ovarian cancer cells Cell proliferation was assayed by MTT assay in seven primary cultures of ovarian cancer cells after 72 h treatment with simvastatin **A.** The IC50 value for primary cultures is shown in **B.** HMGCR protein expression was detected by western blotting in the seven untreated primary cultures of ovarian cancer cells **C, D.** HMGCR expression did not predict sensitivity to simvastatin in ovarian cancer cells.

To expand on our work in established cell lines and ovarian cancer mouse model, we further investigated the effects of simvastatin on cell growth in primary cultures of ovarian cancer cells using the MTT assay. Our results demonstrated that the majority of the primary cultures responded to simvastatin, with 5/7 cases achieving an IC50 (range: 3 to 23 uM) after 72 h of treatment with simvastatin (Fig. 8A and 8B). To further investigate if HMGCR protein expression was associated with sensitivity to simvastatin, we detected the HMGCR protein by Western blot in all of the primary cultures of ovarian cancer cells (Fig. 8C and 8D). The results revealed no correlation between level of HMGCR expression and response to treatment with simvastatin in our primary cultures of ovarian cancer cells.

## DISCUSSION

In this study, we investigated the anti-neoplastic activity of simvastatin in human ovarian cancer cell lines, primary cultures of ovarian cancer cells and an orthotopic mouse model of ovarian cancer. Simvastatin was found to inhibit cellular proliferation, suppress HMGCR enzymatic activity, cause mitochondrial DNA damage, cell cycle arrest, induce apoptosis and cellular stress, and block cellular adhesion and invasion. Moreover, the growth inhibition induced by simvastatin was accompanied by inhibition of the MAPK and AKT/mTOR/S6 pathways. Treatment with simvastatin reduced ovarian tumor weight and volume in mice, which was coincident with a decrease in expression of Ki67, p-pAKT, p-p42/44 and HMGCR, and an increase in cleaved caspase 3. These observations are comparable to recent studies in breast, liver, melanoma and lung cancer, showing promising anti-tumorigenic effects of statins on the growth of cancer *in vitro* and *in vivo* [24–28].

Adhesion and invasion are early steps involved in the metastatic process for ovarian cancer, which has a complex molecular basis that involves adhesion molecules, cell surface receptors, oncogenes, chloride channels, fatty acid synthase, and focal adhesion kinase [29–32]. Several studies have reported that simvastatin inhibits adhesion and invasion in leukemias, hepatocellular cancer, melanoma and endometrial cancer through multiple cell signaling pathways such as the ROCK, MAPK and mTOR pathways [21, 25, 33–35]. Simvastatin has been found to reduce ovarian cancer cell adhesion to peritoneal mesothelial cells through decreased expression of VCAM-1 and β1 integrin *in vitro* [36], suggesting that simvastatin may provide a novel therapeutic approach to the prevention of peritoneal carcinomatosis. Overexpression of VEGF in primary tumor and serum has been associated with poor progression-free survival (PFS) and overall survival for patients with ovarian cancer [37]. Simvastatin treatment has been found to decrease serum VEGF levels in diabetic patients [38] and to reduce intra-tumoral VEGF in mice [39]. Our data finds that simvastatin inhibited adhesion and invasion in two ovarian cancer cell lines and reduced VEGF production in their culture media and cell lysates. In addition, treatment with simvastatin in a mouse model of ovarian cancer also decreased VEGF levels in serum and tumor tissues.

To date, there are few studies that have looked at statin's anti-proliferative effects in gynecologic cancer cells [40]. One study analyzed the effects of lipophilic (simvastatin and lovastatin) *versus* hydrophobic (pravastatin) statins on cell proliferation in gynecologic cancer cell lines, primary cultures of endometrial cancer and normal tissues, and found that lipohillic but not hydrophilic statins induced cell death in a dose and time dependent manner in endometrial, ovarian and cervical cancer cells, whereas little or no toxicity was observed in normal cells [40]. Another study demonstrated that all statins except pravastatin resulted in cell growth arrest in either monolayer cultures or spheroids cultures through activation of the autophagy pathway in seven different ovarian cancer cell lines [3]. In order to clarify the molecular mechanism of simvastatin-induced growth arrest, we comprehensively investigated the effect of simvastatin on the cell cycle, apoptosis and cellular stress. Inhibition of growth induced by simvastatin led to G1 cell cycle arrest and an increase in Annexin V expression in our ovarian cancer cells, which was accompanied by increased intracellular mitochondrial apoptosis, mitochondrial DNA damage and cellular ROS. Similar effects were observed in our orthotopic mouse model of ovarian cancer, which demonstrated that simvastatin blocked tumor growth *via* inhibition of the MAPK and mTOR/S6 pathways and activation of the mitochondrial apoptosis pathway. Increased ROS levels occurred within 24 h after simvastatin treatment, suggesting that redox imbalance is an early event induced by simvastatin in ovarian cancer cells. In fact, higher ROS production induced by simvastatin directly related to increased DNA damage, diminished DNA synthesis and caused cell cycle arrest in breast and liver cancer cells [41, 42].

Statins are known to reduce cell proliferation via down-regulation of critical signaling pathways in a few human cancers, including AKT/mTOR, MAPK, JAK2/STAT3, Ras, NF-κB, JUNK and RhoA/ROCK pathways [9, 10, 43, 44]. In our previous study in simvastatin on endometrial cancer, we found that treatment with simvastatin resulted in inhibition of the MAPK pathway and exhibited differential effects on the AKT/mTOR pathway in the ECC-1 and Ishikawa cells [35]. Similar results have been obtained in renal cancer cells and human myoblasts [44, 45]. The current study shows that simvastatin inhibited the protein expression of phosphorylated p42/44, AKT and S6 in in ovarian cancer cell lines and orthotropic xenografts of serous ovarian cancer, suggesting simvastatin reduced the activity MAPK and AKT/mTOR pathways in ovarian cancer. These results are consistent with previously published data, which confirmed that the MAPK and AKT/mTOR pathways regulated growth inhibition induced by simvastatin *in vitro* and *in vivo*.

We acknowledge that the doses of simvastatin used in our *in vitro* study (IC 50 value, 10 uM for Hey and 8 uM for SKOV3, 3–23 uM for primary culture cells) are supra-therapeutic compared to the doses used in hypercholesterolemia patients. However, the range of doses are very similar to those used in other *in vitro* studies of simvastatin in cancer cells (1–30 uM) [31–34]. The maximum recommended clinical simvastatin dose is 80 mg/day and the maximum tolerated dose of simvastatin is 15 mg/kg/day [46]. The therapeutic dose of simvastatin, 1 mg/kg/day, correlates to a serum level of 0.1 uM [36]. Our *in vivo* study found that simvastatin significantly inhibited the growth of orthotropic xenografts of ovarian cancer at a dose of 3 mg/kg/day without any notable side effects, which is close to therapeutic concentrations clinically. This is a lower dose when compared to previously studied mouse models using 5 mg/kg/day [44].

Many epidemiological studies have shown a correlation between statins use and a relative reduction in the risk of endometrial, colorectal, gastric, and hepatocellular cancer [18, 47–51]. Currently, statins are being studied clinically for use in the prevention and treatment of cancer and as an adjuvant therapy in combination with chemotherapeutic agents. Two colorectal cancer studies reported a 17–51% reduction in colorectal cancer risk with simvastatin [50, 51]. Improved overall survival was seen in patients who used statins one year prior to the diagnosis of endometrial and ovarian cancers [18]. Reduced rates of advanced disease have been found among prostate cancer patients taking statins at the time of prostatectomy [52]. A clinical trial study comparing advanced liver cancer patients taking pravastatin *versus* a placebo showed increased survival amongst those patients who had been given the statin [53]. Breast cancer patients who were given atorvastatin two weeks prior to surgery showed anti-proliferative effects, with a decrease in Ki-67 staining in those tumors that were HMGCR positive [54]. Although statins used as a single agent have shown some promise, statins may also be useful in combination with other cytotoxic agents. A phase II study using simvastatin plus irinotecan, 5-fluorouracil, and leucovorin (FOLFIRI) as first-line chemotherapy in metastatic colorectal cancer showed that time to progression was prolonged, and no additional adverse effects were seen with the addition of simvastatin [17]. Thus, given promising epidemiological and pre-clinical data, statins may be useful in the treatment and possibly prevention of many cancers, including ovarian cancer. Given that obese patients with ovarian cancer often suffer from co-morbidities such as hypercholesterolemia and diabetes, statins may be a particularly innovative treatment and prevention strategy in this high risk patient population.

## MATERIALS AND METHODS

### Cell culture and reagents

Hey and SKOV3 ovarian cancer cell lines were used in these experiments. Hey cells were maintained in RPMI 1640 with 5% fetal bovine serum. SKOV3 cells were grown in DMEM/F12 with 10% fetal bovine serum. Simvastatin, MTT (3-(,5-dimethylthiazol-2-yl)-2,5-diphenyltetrazolium bromide) and RNase A were purchased from Sigma (St. Louis, MO). The anti-phosphorylated-AKT, anti-pan-AKT, anti-phosphorylated-p42/44, anti-pan-p42/44, anti-phosphorylated-S6, anti-pan-S6, anti-cleaved caspase 3 and anti-BCL-2 antibodies were purchased from Cell Signaling (Beverly, MA). The anti-HMGCR antibody was from Santa Cruz (Dallas, Texas). Enhanced chemiluminescence Western blotting detection reagents were purchased from Amersham (Arlington Heights, IL). All other chemicals were purchased from Sigma.

### Cell proliferation assays

The Hey and SKOV3 cells were plated and grown in 96-well plates at a concentration of 4000 cells/well for 24 h. Cells were subsequently treated with varying doses of simvastatin for 72 h. MTT (5 mg/ml) was added to the 96-well plates at 5 μl/well, followed by an additional hour of incubation. The MTT reaction was terminated through the addition of 100 μl of DMSO. The results were read by measuring absorption at 570 nm with a Microplate Reader (Tecan, Morrisville, NC). The effect of simvastatin was calculated as a percentage of control cell growth obtained from DMSO treated cells grown in the same 96-well plates. Each experiment was performed in triplicate to assess for consistency of results.

### Apoptosis assay

Simvastatin induced apoptosis was detected with the Annexin V FITC kit (Biolegend, San Diego, CA) on the Cellometer (Nexelom, Lawrence, MA). Briefly, 2 × 10^5^ cells/well were seeded into 6-well plates, incubated overnight and then treated with simvastatin at different doses for 24 h. The cells were then collected, washed with PBS and resuspended in 100 ul binding buffer. Subsequently, 1 ul of annexin V-FITC (100 ug/ml) and 0.5 ul of propidium iodide (2 mg/ml) were added in the binding buffer and placed in the dark for 15 minutes. The samples were immediately measured by Cellometer. The results were analyzed by FCS4 express software (Molecular Devices, Sunnyvale, CA). All experiments were performed in triplicate to assess for consistency of response.

### Cell cycle assay

The effects of simvastatin on cell cycle progression were measured by Cellometer. Briefly, 2.5 × 10^5^ cells/well were seeded into 6-well plates, incubated overnight and then treated with simvastatin at different concentrations for 24 h. The cells were harvested and washed with phosphate buffered saline (PBS). The pellet was re-suspended and fixed in 90% pre-chilled methanol and stocked overnight at −20°C. The cells were then washed with PBS again and resuspended in 50 μl RNase solution (250 ug/ml) and 10 mM EDTA for 30 minutes. Finally, 50 μl staining solution [containing 2 mg/ml PI (Biotium, Hayward, MA), 0.1 mg/ml Azide (Sigma-Aldrich), and 0.05% Triton X-100 (Sigma-Aldrich)] was added, and the final mixture was incubated for 15 minutes in the dark before being analyzed on Cellometer. The measured results were analyzed using the FCS4 express software (Molecular Devices, Sunnyvale, CA). Cell cycle analysis assay was performed in duplicate.

### Adhesion assay

Each well in a 96-well plate was coated with 100 ul laminin-1 (10 ug/ml) and incubated at 37°C for 1 h. The fluid was then aspirated and 200 ul blocking buffer was added to each well for 45–60 min at 37°C. The wells were then washed with PBS, and the plate was allowed to chill on ice. To each well, 2.5 × 10^3^ cells were added with PBS and varying concentrations of simvastatin directly. The plate was then allowed to incubate at 37°C for 2 h. After this period, the medium was aspirated, and the cells were fixed by directly adding 100 ul of 5% glutaraldehyde and incubating for 30 min at room temperature. Adhered cells were then washed with PBS and stained with 100 ul of 0.1% crystal violet for 30 min. The cells were then washed repeatedly with water, and 100 ul of 10% acetic acid was added to each well to solubilize the dye. After 5 minutes of shaking, the absorbance was measured at 570 nm using a microplate reader from Tecan (Mannedorf, Switzerland). All experiments were performed in duplicate to assess for consistency of response.

### Invasion assay

Cell invasion assays were performed using 96-well HTS transwells (Corning Life Sciences, Wilmington, NC) coated with 1X BME (Trevigen, Gaithersburg, Maryland). Starved (serum-free media for 12 h) Hey and SKOV3 cells (50,000 cells/well) were seeded for 12 h in the upper chambers of the wells in 50 μl FBS-free medium, and the lower chambers were filled with 150 μl regular medium with simvastatin. The plate was incubated for 24 h at 37°C to allow invasion into the lower chamber. After washing the upper and lower chambers with PBS once, 100 ul Calcein AM solution was added into the lower 37°C chamber and incubated for 30–60 min. The lower chamber plate was measured by the plate reader for reading fluorescence at EX/EM 485/520 nM. All experiments were performed in duplicate to assess for consistency of response.

### Reactive oxygen species (ROS) assay

ROS generation was assessed using the ROS-sensitive fluorescence indicator, DCFH-DA. To determine intracellular ROS scavenging activity, Hey and SKOV3 cells (1.0 × 10^4^ cells/well) were seeded in black 96-well plates. After 24 h, the cells were treated with simvastatin for 18 h to induce ROS generation. After the cells were incubated with DCFH-DA (20 μM) for 30 minutes, the fluorescence intensity was measured at an excitation wavelength of 485 nm and an emission wavelength of 530 nm using a fluorescence microplate reader. All experiments were performed in duplicate to assess for consistency of response.

### DNA damage assay

High-molecular-weight DNA was isolated using a QIAamp DNA mini kit (QIAGEN, Venlo, Limberg) following the recommended protocol. The concentration of total cellular DNA was determined by using the Nanodrop (Tecan, Mannedorf, Switzerland). Quantitative PCR (qPCR) assays were performed as previously described with minor modifications [18, 21]. The primers for large fragments of the mtDNA (8.9 kb) are forward 5′-TCA AAG CCT CCT TAT TCG AGC CGA-3′, reverse 5′–TTT CATCAT GCG GAG ATG TTG GAT GG-3′, and primers for small mtDNA (221 bp) fragment are forward 5′–CCC CAC AAA CCC CAT TAC TAA ACC CA-3′, reverse 5′ – TTT CATCAT GCG GAG ATG TTG GAT GG - 3′. A total volume of 50 μl was used in PCRs containing: 15 ng of template DNA, 5 pmol of each primer, 10X mix buffer and 2.5 units of recombinant Taq DNA polymerase High Fidelity (Invitrogen, Carlsbad, CA). A quantitative control using half the concentration of control template DNA was included in each set of PCR reactions. Small fragments (211 bp) of the mtDNA were also amplified for internal controls, respectively. The internal controls were used to normalize the results obtained from the large fragments and to monitor the mitochondrial copy number. The thermal cycling conditions were as follows: 95°C for 3 min, followed by 19 cycles of 94°C for 1 min, 64°C for 1 min and 68°C for 9 min, primer extension at 72°C for 3 min at the end of these cycles. Every sample was tested in triplicate. qPCR products were quantitated using the Quant-iT™ dsDNA High Sensitivity Assay Kit. The average lesion frequency per each fragment was calculated by using the Poisson equation [19]. This experiment was done in duplicate to assess for consistency of response.

### VEGF assay

VEGF levels of media, serum and ovarian cancer tissue homogenates were measured by the enzyme-linked immunoassay method using commercially available kits (human or mouse VEGF ELISA kit, RayBiotech, Inc., Norcross, GA,). The assay was conducted in triplicate.

### Blood cholesterol

Blood cholesterol from the mice was measured by an automated blood chemical analyzer (Ortho Clinical Diagnostic Inc, Rochester, NY) in the UNC-CH Animal Facility Laboratory, Department of Pathology, UNC, Chapel Hill.

### Western immunoblotting

Hey and SKOV3 cells were plated at 2–4 × 10^5^ cells/well in 6 well plates in their appropriate media and were treated with simvastatin for 24 h in 0.5% stripped serum. Cell lysates were prepared in RIPA buffer (1% NP40, 0.5 sodium deoxycholate and 0.1% SDS) plus PhosStop. Equal amounts of protein were separated by gel electrophoresis and transferred onto a PVDF membrane. The membrane was blocked with 5% nonfat dry milk and then incubated with a 1:1000 dilution of primary antibody overnight at 4°C. The membrane was then washed and incubated with a secondary peroxidase conjugated antibody for 1 hour after washing. Antibody binding was detected using an enhanced chemiluminescence detection buffer and the Alpha Innotech imaging system (San Leandro, CA). After developing, the membrane was re-probed using antibody against α-tubulin or β-actin. Each experiment was repeated three times to assess for consistency of results.

### Ovarian cancer tissue sample collection and primary cell culture

Seven tumor specimens were sampled from patients undergoing surgery for ovarian cancer at the University of North Carolina at Chapel Hill. The protocol was reviewed and exemption granted by the Institutional Review Board at the University. Freshly obtained tissues were washed three times with Hank's Buffered Salt Solution (HBSS), and then gently minced by scissors in DMEM/F12 medium containing 10% fetal bovine serum (FBS). Primary cultures were performed as described [23]. 2 × 10^4^ primary culture cells/well were seeded into 96-well plates and incubated for 24 h before treatment with simvastatin. Cell proliferation was measured by MTT assay 72 h after treatment.

### Orthotropic xenografts of serous ovarian cancer

The K18-gT121+/− p53fl/fl Brca1fl/fl (KpB) mouse model is a unique serous ovarian cancer mouse model that specifically and somatically deletes the tumor suppressor genes, Brca1 and p53, and inactivates the retinoblastoma (Rb) proteins in adult ovarian surface epithelial cells (KpB mouse model) [22]. As an extension of this model, we have established an ovarian tumor cell line from one of the KpB mice (M909). Upon re-injection of these tumor cells into the ovarian bursa of female mice, we have developed a more aggressive variant of the KpB model [23]. For the evaluation of simvastatin's *in vivo* effects, M909 cells (1 × 10^6^ cells/5 μl) were injected into the left side of the ovarian bursa of 6–8 week old mice. All mice were handled according to protocols approved by UNC-CH Institutional Animal Care and Use Committee (IACUC). Twenty two mice were injected with the M909 cells and then randomly divided into the control and simvastatin groups. The simvastatin treatment (intraperitoneal injection, 3 mg/kg/day) was initiated twelve days after the injection, and tumor size was checked twice a week using palpation until tumors had grown to a size amenable to caliper measurement. Tumor volume was calculated using the following equation (width^2^ × length)/2. All mice were euthanized after four weeks of simvastatin or placebo treatment. Tumor tissue and blood samples were collected for immunohistochemical staining and VEGF assay.

### Immunohistochemistry

Five micrometer paraffin sections prepared from the transgenic mice were used for immunohistochemical analysis. Staining procedures were performed at Histology Research Core Facility at UNC. The following primary antibodies were used: Ki-67, HMGCR, cleaved caspase 3, phos-AKT and phos-p42/44 expression. Further processing was carried out using ABC-Staining Kits (Vector Labs, Burlingame, CA) and hematoxylin. Immunochemistry slides were scanned, analyzed and scored by Aperio and ImageScope software (Vista, CA).

### Statistical analysis

Results were compared by Student's *t* test and data were expressed as mean ± S.E. Statistical significance was defined to be *p* < 0.05.

## SUPPLEMENTARY FIGURE


