# Supplementary material for: The HMG-CoA reductase inhibitor, simvastatin, exhibits anti-metastatic and anti-tumorigenic effects in ovarian cancer
Source: Oncotarget. 2015 Oct 16;7(1):946–60. doi: 10.18632/oncotarget.5834 (PMC4808044; doi:10.18632/oncotarget.5834)
Supplement: Supplementary file 1 [file oncotarget-07-0946-s001.pdf]

## SUPPLEMENTARY FIGURE

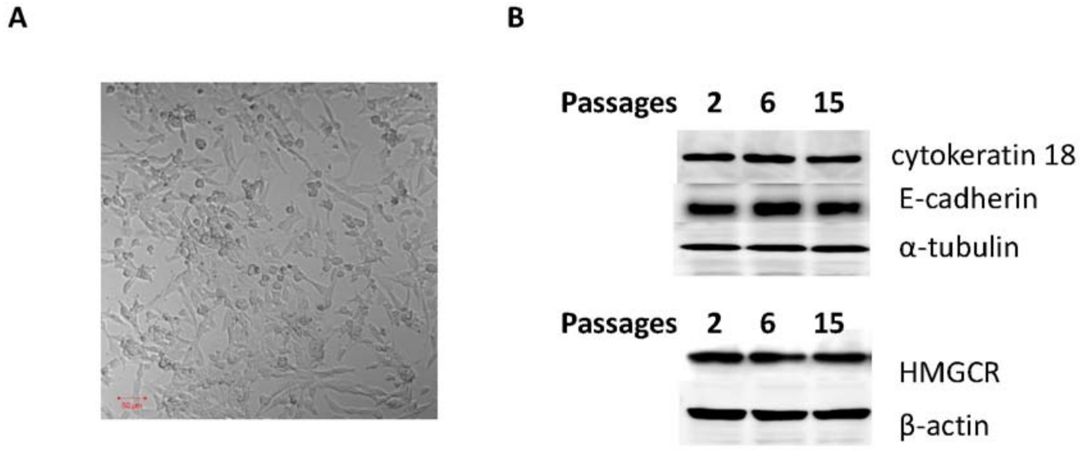


**Supplementary Figure S1: Cellular morphology and expression of keratin, E-cadherin and HMGCR in M909 cells.** Morphological appearance of the M909 at passage 6 was seen in Fig A. Western blotting showed that cytokeratin 18, E-cadherin and HMGCR were positive in M909 cells at passage 2, 6 and 15 (Fig. B).
